# PLXNC1 interference alleviates the inflammatory injury, apoptosis and extracellular matrix degradation of IL-1β-exposed chondrocytes via suppressing GRP78 expression

**DOI:** 10.1186/s13018-023-04207-4

**Published:** 2023-10-18

**Authors:** Nan Meng, Lingwei Mao, Qinyi Jiang, Jishan Yuan, Linjuan Liu, Lei Wang

**Affiliations:** 1 Department of Orthopedics, The Affiliated People’s Hospital with Jiangsu University, 8 Dianli Road, Runzhou District, Zhenjiang City, 212002 Jiangsu Province China; 2 Department of Stomatology, The Affiliated Hospital with Jiangsu University, 8 Jiefang Road, Jingkou District, Zhenjiang City, 212002 Jiangsu Province China

**Keywords:** PLXNC1, GRP78, ECM degradation, Chondrocytes, OA

## Abstract

**Background:**

Osteoarthritis (OA) is a frequently encountered debilitating joint disorder. Whether plexin C1 (PLXNC1) is implicated in OA is far from being investigated despite its well-documented pro-inflammatory property in human diseases. The goal of this study is to expound the specific role of PLXNC1 in OA and elaborate the probable action mechanism.

**Methods:**

Firstly, PLXNC1 expression in the cartilage tissues of patients with OA was examined with GEO database. In interleukin-1beta (IL-1β)-induced OA cell model, RT-qPCR and western blotting tested the expression of PLXNC1, glucose-regulating protein 78 (GRP78) and extracellular matrix (ECM) degradation-related factors. Cell viability and inflammation were respectively judged by CCK-8 assay and RT-qPCR. TUNEL and western blotting estimated cell apoptosis. The potential binding between PLXNC1 and GRP78 was corroborated by Co-IP assay. Western blotting also tested the expression of endoplasmic reticulum stress (ERS)-associated proteins.

**Results:**

As it turned out, PLXNC1 expression was elevated in the cartilage tissues of patients with OA and IL-1β-treated chondrocytes. When PLXNC1 was depleted, the viability injury, inflammation, apoptosis and ECM degradation of chondrocytes exposed to IL-1β were obstructed. Besides, GRP78 bond to PLXNC1 in IL-1β-treated chondrocytes. The ascending GRP78 expression in the chondrocytes exposed to IL-1β was depleted after PLXNC1 was silenced. Meanwhile, the impacts of PLXNC1 deficiency on the viability, inflammatory response, apoptosis, ECM degradation as well as ERS in IL-1β-exposed chondrocytes were abolished by GRP78 up-regulation.

**Conclusion:**

In summary, PLXNC1 silencing might interact with and down-regulate GRP78 to mitigate the apoptosis, inflammation, and ECM degradation of IL-1β-insulted chondrocytes in OA.

## Introduction

Osteoarthritis (OA) is a frequently occurring joint disease with articular cartilage damage involving the whole joint, the clinical manifestations of which mainly include joint pain, stiffness and dysfunction [1, 2]. The susceptible factors of OA include obesity, aging and overload exercise [3, 4]. As unveiled by the epidemiological survey, the global case rate of OA has reached up to 16% in 2017 and the prevalence rises annually with an aging population [5]. Also, OA is extensively deemed as a leading contributor to disability worldwide [3]. Considering that there are no effective therapeutic modalities capable of reversing or terminating the process of OA, more efforts need to be made to seek for reliable treatment methods. Meanwhile, the complicated pathogenesis of OA remains an obstacle in cartilage degeneration in spite of the promise raised by stem cells in cartilage repair [6].

Plexin C1 (PLXNC1) located on chromosome 12 is a member of Plexin family composed of transmembrane proteins [7]. PLXNC1 which was first described in the optic tectum participates in neuron cell adhesion and migration as a target receptor of Semaphorin 7A [8]. In addition to the role in nervous system, there is considerable evidence in favor of the involvement of PLXNC1 in human diseases through regulating immunological response. For example, knockdown of PLXNC1 plays the suppressive role in hepatic ischemia–reperfusion injury and lung injury through reducing inflammatory response [8, 9]. Nevertheless, the effects of PLXNC1 on the biological processes of OA remain to be elaborated.

After potential interacting partners were obtained according to Biogrid database, HDOCK server predicted that PLXNC1 might bind to glucose-regulating protein 78 (GRP78). GRP78 is a multi-functional protein which is primarily expressed in the lumen of the endoplasmic reticulum [10, 11]. Existing evidence has proposed that GRP78 extensively participates in human malignancies and diseases via mediating endoplasmic reticulum stress and unfolded protein response as a molecular chaperone in endoplasmic reticulum [10–12]. Moreover, GRP78 expression is increased in cartilages as OA progresses [13] and GRP78 interference suppresses oxidative stress in chondrocytes [14].

Accordingly, this study is conducted with the aim of shedding light on the role of PLXNC1 in OA and ascertaining the regulatory mechanism between PLXNC1 and GRP78.

## Materials and methods

### Bioinformatics tools

PLXNC1 expression in OA tissues was examined in dataset GSE168505 downloaded from GEO database (https://www.ncbi.nlm.nih.gov/geo/). Potential interacting partners were obtained according to Biogrid database (https://thebiogrid.org/) and HDOCK server (http://hdock.phys.hust.edu.cn/) predicted the potential interaction between PLXNC1 and GRP78 [15].

### Cell culture, treatment and transfection

Dulbecco’s modified Eagle medium (DMEM; MesGenBiotech, Shanghai, China) was applied for the cultivation of human chondrocyte cell line CHON-001 from American Type Culture Collection (ATCC, Rockville, MD, USA), accompanied with 10% fetal bovine serum (FBS; MesGenBiotech, Shanghai, China) as well as 1% antibiotics (MesGenBiotech, Shanghai, China) under 5% CO_2_ at 37 °C. Ascending doses of IL-1β (2.5, 5 and 10 ng/ml; MesGenBiotech, Shanghai, China) were to treat cells for 72 h, and 10 ng/ml IL-1β was eventually selected for the construction of OA cell model [16].

The small interfering RNAs (siRNAs) specific for PLXNC1 (SiRNA-PLXNC1-1/2) and the blank control (SiRNA-NC) were ordered from Guangzhou Ribobio Co., Ltd. GRP78 overexpression vector (Ov-GRP78) was developed by Sino Biological Inc. (Beijing, China) via inserting the prepared GRP78 cDNA into the pcDNA3.1(+) plasmid, referring to the empty vector as Ov-NC. Aforementioned plasmids were delivered into CHON-001 cells by Lipofectamine 2000 (Invitrogen) as per the manufacturer’s recommendation.

### Cell counting kit-8 (CCK-8)

Briefly, ascending doses of IL-1β (2.5, 5 and 10 ng/ml) were to treat cells (1 × 10^5^ cells/well) seeded in a 96-well plate at 37 °C. Prior to the examination of OD450nm value with a microplate reader (Hidex, Turku, Finland), 10 μl CCK-8 solution (Wuhan Elabscience Biotechnology Co., Ltd.) was supplemented for extra 2 h in conformity to the product manual.

### Reverse transcription-quantitative PCR (RT-qPCR)

The synthesis of cDNA was performed with the aid of SureScript™ First-Strand cDNA Synthesis Kit (GeneCopoeia, USA) following the preparation of total RNA from cells adopting RNAzol® RT RNA Isolation Reagent (GeneCopoeia, USA). Relative gene expression was calculated by virtue of 2^−ΔΔCq^ method following PCR amplification with BlazeTaq™ SYBR® Green qPCR mix (GeneCopoeia, USA), viewing GAPDH as the loading control. The primer sequences used are as follows: PLXNC1, forward, 5′-AGAGTCCAACCAATCGCATCA-3′ and reverse, 5′-AGTCCTGTTCATTACCACGGT-3′; GRP78, forward, 5′-GAACGTCTGATTGGCGATGC-3′ and reverse, 5′-GAGTCGAGCCACCAACAAGA-3′; tumor necrosis factor alpha (TNFα), forward, 5′-CTGGGCAGGTCTACTTTGGG-3′ and reverse, 5′-CTGGAGGCCCCAGTTTGAAT-3′; interleukin-1 beta (IL-1β), forward, 5′-CCAAACCTCTTCGAGGCACA-3′ and reverse, 5′-AGCCATCATTTCACTGGCGA-3′; interleukin-6 (IL-6), forward, 5′-GTCCAGTTGCCTTCTCCCTGG-3′ and reverse, 5′-CCCATGCTACATTTGCCGAAG-3′; matrix metallopeptidase 3 (MMP3), forward, 5′-AGTCTTCCAATCCTACTGTTGCT-3′ and reverse, 5′-TCCCCGTCACCTCCAATCC-3′; matrix metallopeptidase-13 (MMP-13), forward, 5′-ACTGAGAGGCTCCGAGAAATG-3′ and reverse, 5′-GAACCCCGCATCTTGGCTT-3′; collagen II, forward, 5′-CTTCCCCCTCCTGCTCCAAG-3′ and reverse, 5′-CTGGGCAGCAAAGTTTCCAC-3′; a disintegrin and metalloproteinase with thrombospondin motifs type 4 (ADAMTS-4), forward, 5′-GGAAATTCAGATGTGGTACTGCC-3′ and reverse, 5′-CGCATTAGGGCAGAGAGGAG-3′; a disintegrin and metalloproteinase with thrombospondin motifs type 5 (ADAMTS-5), forward, 5′-ACAAGAGCCTGGAAGTGAGC-3′ and reverse, 5′-TTGGACCAGGGCTTAGATGC-3′; and aggrecan, forward, 5′-ACTCTGGGTTTTCGTGACTCT-3′ and reverse, 5′-ACACTCAGCGAGTTGTCATGG-3′.

### Western blotting

The collection of total protein was implemented using RIPA lysis buffer (Hangzhou Fude Biological Technology Co., Ltd.). Following SDS-PAGE electrophoresis, the membranes containing resolved proteins were subjected to blockade of non-specific binding in 5% non-fat milk and immunoblotted with primary antibodies at 4 °C overnight, prior to being probed with HRP-conjugated secondary antibody (ab6721; 1/2000; Abcam). The blots were visualized by the ECL reagent (Hangzhou Fude Biological Technology Co., Ltd.), and the gray analysis was implemented with ImageLab4.0 software. PLXNC1 (cat. no. GTX34127; 1/1000; GeneTex), GRP78 (cat. no. ab108615; 1/1000; Abcam), B cell lymphoma-2 (Bcl-2; cat. no. ab182858; 1/2000; Abcam), Bcl-2-associated X (Bax; cat. no. ab32503; 1/1000; Abcam), cleaved caspase 3 (cat. no. ab32042; 1/500; Abcam), MMP3 (cat. no. ab52915; 1/1000; Abcam), MMP-13 (cat. no. ab39012; 1/3000; Abcam), collagen II (cat. no. ab188570; 1/1000; Abcam), ADAMTS-4 (cat. no. ab185722; 1/1000; Abcam), ADAMTS-5 (cat. no. ab41037; 1/250; Abcam), aggrecan (cat. no. GT1267; 1/1000; GeneTex), tubulin (cat. no. ab6046; 1/500; Abcam), activating transcription factor 4 (ATF4; cat. no. ab184909; 1/1000; Abcam), phosphorylated inositol requiring enzyme 1 alpha (p-IRE1α; cat. no. ab124945; 1/1000; Abcam), IRE1α (cat. no. ab37073; 1/1000; Abcam), tumor necrosis factor receptor-associated factor 2 (TRAF2; cat. no. ab126758; 1/1000; Abcam), apoptosis signal-regulating kinase 1 (ASK1; cat. no. ab45178; 1/1000; Abcam) and GAPDH (cat. no. ab9485; 1/2500; Abcam) primary antibodies were utilized here.

### Terminal deoxynucleotidyl transferase dUTP nick end (TUNEL)

Prior to the assay, CHON-001 cells were treated by 10 ng/ml IL-1β for 72 h. Following PBS washing in the harvested CHON-001 cells, 15 min of immobilization with 4% paraformaldehyde (Shanghai Yeasen Biotechnology Co., Ltd.) was performed at room temperature. Subsequently, prior to DAPI staining, 50 μl TUNEL kit (Shanghai Yeasen Biotechnology Co., Ltd.) was added for 1 h of incubation at 37 °C. Images were acquired under a fluorescence microscope (Olympus).

### Co-immunoprecipitation (Co-IP) assay

Co-IP assay was conducted with the adoption of Co-IP kit (ACE Biotechnology, Nanjing, China). In short, the acquired cell lysates by centrifugation (14,000 rpm, 4 °C, 15 min) were treated by 2 μg of PLXNC1 (cat. no. sc-390216; Santa Cruz Biotechnology) or GRP78 antibodies (cat. no. sc-13539; Santa Cruz Biotechnology) or goat anti-mouse IgG (cat. no. ab6789; Abcam), followed by mixture with protein A/G beads (Shanghai Yeasen Biotechnology Co., Ltd.). Analysis of PLXNC1 and GRP78 enrichment was implemented by western blotting.

### Statistical analyses

The values were given as mean ± standard deviation (SD) adopting GraphPad Prism 8 software (GraphPad Software, Inc.). Observed differences were viewed statistically significant at *p* < 0.05 with the employment of Student’s *t* test or one-way ANOVA followed by Turkey’s test.

## Results

### PLXNC1 expression is augmented in the cartilage tissues of patients with OA and IL-1β-treated CHON-001 cells

**Fig. 1:**
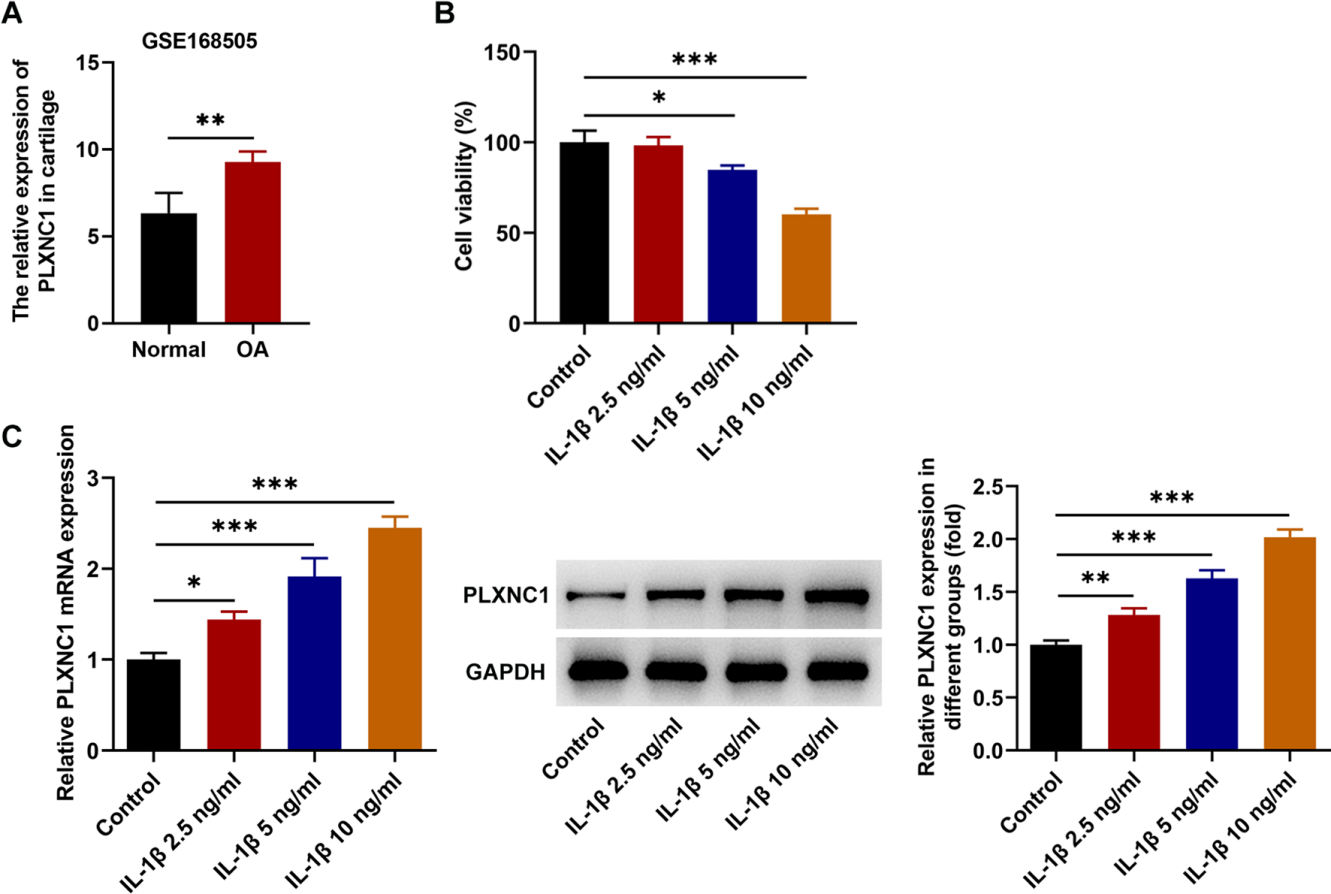
PLXNC1 expression is augmented in the cartilage tissues of patients with OA and IL-1β-treated CHON-001 cells. **A** GEO database examined PLXNC1 expression in the cartilage tissues of patients with OA. CHON-001 cells were treated by ascending doses of IL-1β (2.5, 5 and 10 ng/ml) for 72 h. **B** CCK-8 assay judged CHON-001 cell viability. **C** RT-qPCR and western blotting tested PLXNC1 expression in IL-1β-challenged CHON-001 cells. *P < 0.05; **P < 0.01; and ***P < 0.001. *PLXNC1* plexin C1; *IL-1β* interleukin-1beta

To specify the role of PLXNC1 in OA, PLXNC1 expression was tested. Through dataset GSE168505 from GEO database, it was noted that PLXNC1 expression was markedly increased in the cartilage tissues of patients with OA (Fig. 1A). As exposed by CCK-8 assay, when treated with 5 and 10 ng/ml of IL-1β, CHON-001 cell viability was dose-dependently declined and no apparent alternations were observed when treated with 2.5 ng/ml of IL-1β relative to the control group (Fig. 1B). Thereafter, 10 ng/ml of IL-1β was selected for the induction of OA cell model for its prominently inhibitory role in cell viability. Moreover, RT-qPCR and western blotting analyzed that PLXNC1 expression was also elevated in CHON-001 cells exposed to ascending doses of IL-1β (Fig. 1C). Accordingly, PLXNC1 was overexpressed in the cartilage tissues of patients with OA and OA cell model.

### PLXNC1 absence alleviates IL-1β-induced viability injury and inflammatory response in CHON-001 cells

**Fig. 2:**
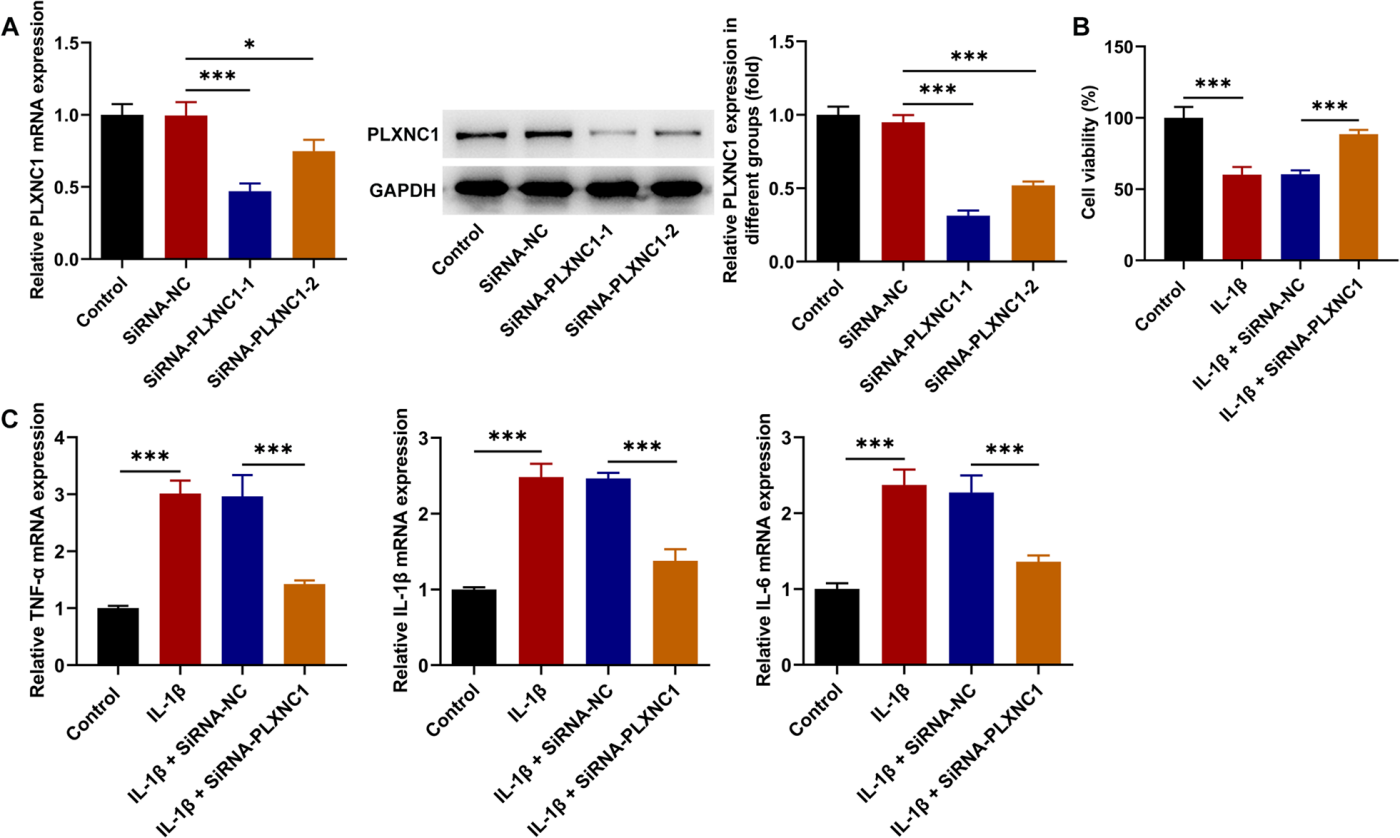
PLXNC1 absence alleviates IL-1β-induced viability injury and inflammatory response in CHON-001 cells. **A** RT-qPCR and western blotting tested the transfection efficiency of PLXNC1 interference plasmids. CHON-001 cells were treated by ascending doses of IL-1β (10 ng/ml) for 72 h and subsequently transfected with SiRNA-NC and SiRNA-PLXNC1. **B** CCK-8 assay judged CHON-001 cell viability. **C** RT-qPCR examined the levels of inflammatory factors including TNFα, IL-1β and IL-6. *P < 0.05 and ***P < 0.001. *PLXNC1* plexin C1, *IL-1β* interleukin-1beta, *TNFα* tumor necrosis factor alpha, *IL-6* interleukin-6

To ascertain the involvement of PLXNC1 in the process of OA, PLXNC1 was remarkably knocked down following transduction of SiRNA-PLXNC1-1/2 plasmids. Further, SiRNA-PLXNC1-1 presented a more notable interference efficacy, hence being applied to the ensuing assays (Fig. 2A). The experimental data from CCK-8 assay elucidated that the deteriorated viability of IL-1β-treated CHON-001 cells was improved following silencing of PLXNC1 (Fig. 2B). Concurrently, it was discovered from RT-qPCR analysis that IL-1β exposure elevated TNFα, IL-1β and IL-6 levels, which were then lessened again by PLXNC1 deficiency (Fig. 2C). To conclude, PLXNC1 down-regulation protected against IL-1β-elicited CHON-001 cell viability injury and inflammation.

### PLXNC1 depletion hampers the apoptosis and ECM degradation of IL-1β-challenged CHON-001 cells

**Fig. 3:**
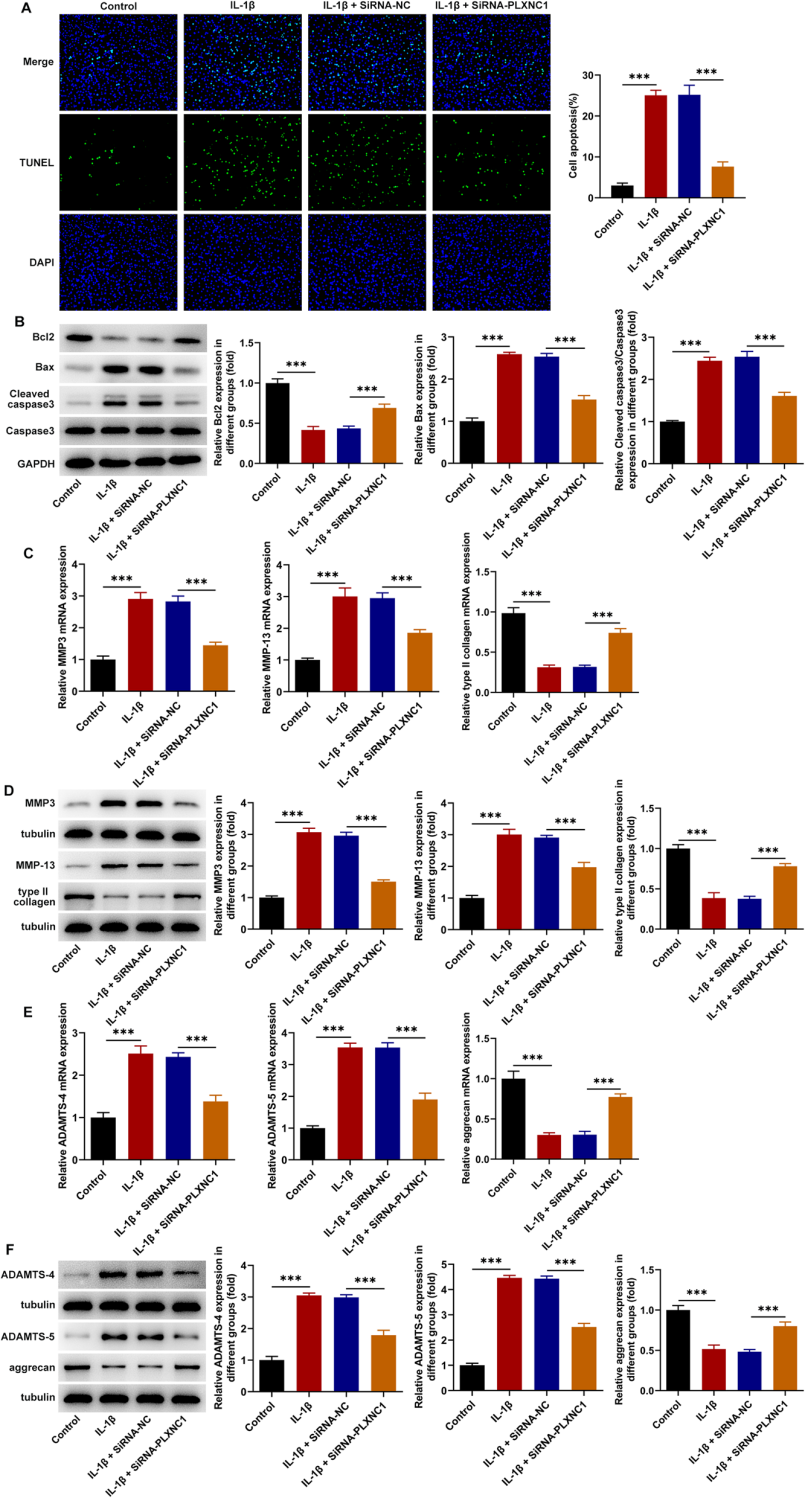
PLXNC1 depletion hampers the apoptosis and ECM degradation of IL-1β-challenged CHON-001 cells. CHON-001 cells were treated by ascending doses of IL-1β (10 ng/ml) for 72 h and subsequently transfected with SiRNA-NC and SiRNA-PLXNC1. **A** TUNEL assay measured apoptosis. **B** Western blot analysis of the expression of apoptosis-associated proteins including Bcl-2, Bax, cleaved caspase 3 and caspase 3. **C**–**F** RT-qPCR and western blotting tested the expression of ECM degradation-associated proteins including MMP3, MMP-13, type II collagen, ADAMTS-4, ADAMTS-5 and aggrecan. ***P < 0.001. *PLXNC1* plexin C1, *IL-1β* interleukin-1beta, *Bcl-2* B cell lymphoma-2, *Bax* Bcl-2-associated X, *MMP3* matrix metallopeptidase 3, *MMP-13* matrix metallopeptidase-13, *ADAMTS-4* a disintegrin and metalloproteinase with thrombospondin motifs type 4, *ADAMTS-5* a disintegrin and metalloproteinase with thrombospondin motifs type 5

Moreover, the results of TUNEL assay manifested that PLXNC1 absence noticeably diminished the elevated apoptotic rate of CHON-001 cells upon exposure to IL-1β (Fig. 3A). Further, western blotting implied that IL-1β challenge resulted in the decrease on Bcl-2 expression and the increase on Bax and cleaved caspase 3/caspase 3 expression, while PLXNC1 interference conversely raised Bcl-2 expression and cut down Bax and cleaved caspase 3/caspase 3 expression in IL-1β-treated CHON-001 cells (Fig. 3B). Additionally, the fortified MMP3, MMP-13, ADAMTS-4, ADAMTS-5 expression and the declined type II collagen, aggrecan expression in IL-1β-treated CHON-001 cells were all restored when PLXNC1 was down-regulated (Fig. 3C–F). Above all, PLXNC1 inhibition obstructed IL-1β-triggered CHON-001 cell apoptosis and ECM degradation.

### PLXNC1 interacts with GRP78 in IL-1β-treated CHON-001 cells

**Fig. 4:**
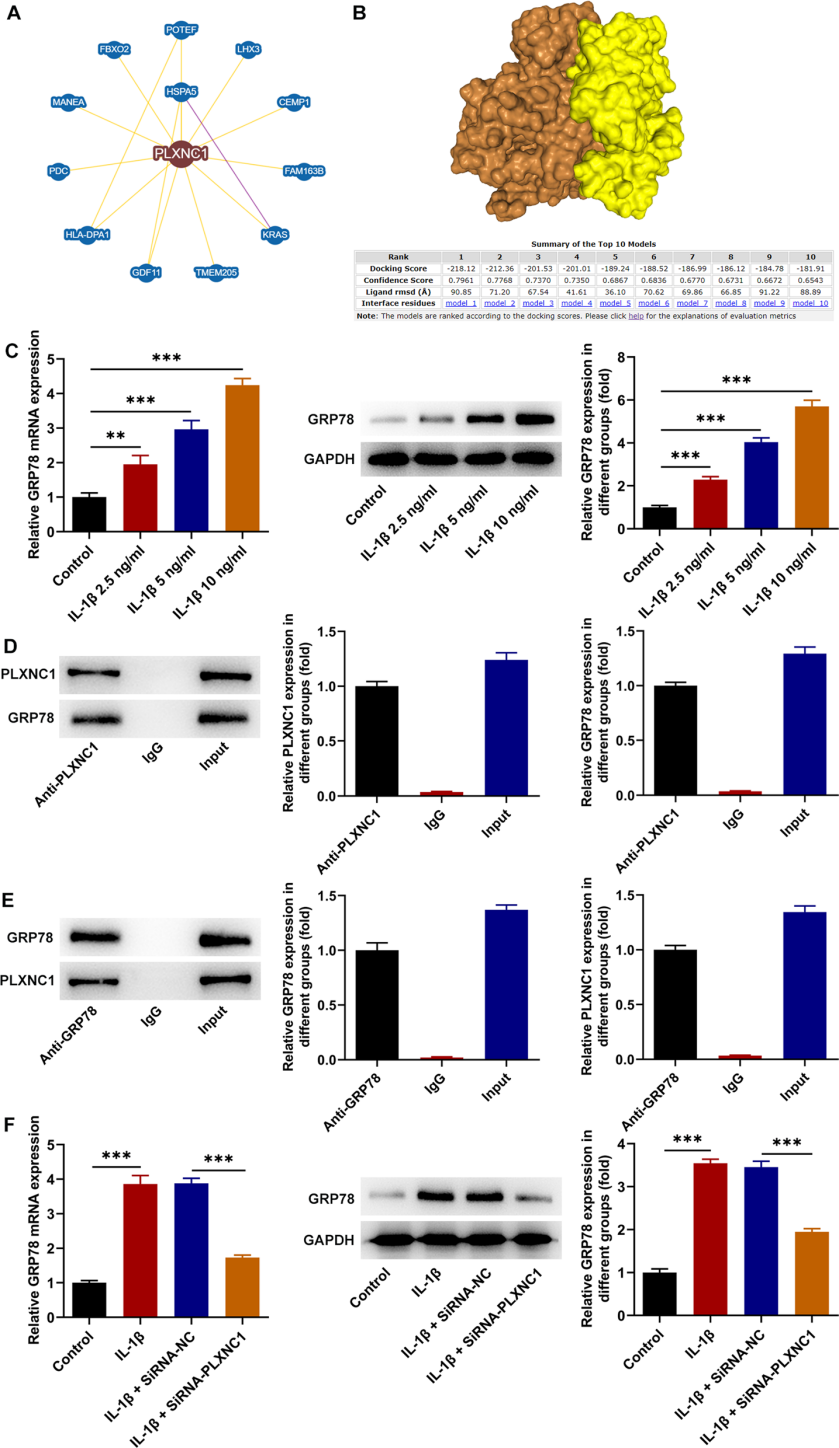
PLXNC1 interacts with GRP78 in IL-1β-treated CHON-001 cells. **A** The potential interacting partners of PLXNC1 were obtained via Biogrid database. **B** The possible interaction between PLXNC1 and GRP78 was predicted by HDOCK server. PLXNC1 and GRP78 were colored in brown and yellow, respectively. **C** CHON-001 cells were treated by ascending doses of IL-1β (2.5, 5 and 10 ng/ml) for 72 h. RT-qPCR and western blotting tested GRP78 expression in IL-1β-challenged CHON-001 cells. **D** and **E** Co-IP assay corroborated the binding between PLXNC1 and GRP78. **F** CHON-001 cells were treated by ascending doses of IL-1β (10 ng/ml) for 72 h and subsequently transfected with SiRNA-NC and SiRNA-PLXNC1. RT-qPCR and western blotting tested GRP78 expression following PLXNC1 absence. **P < 0.01 and ***P < 0.001. *PLXNC1* plexin C1, *IL-1β* interleukin-1beta, *GRP78* glucose-regulating protein 78

As depicted in Fig. 4A, the potential interacting partners of PLXNC1 were obtained via Biogrid database. A total of 12 proteins were ultimately identified as interacting with PLXNC1 by high throughput and physical interactions after the search area was restricted and the minimum 3 evidence was chosen. Following validation, the heat shock protein GRP78, which is mostly localized at the ER and is encoded by HSPA5, was chosen for more examination. Also, the possible interaction between PLXNC1 and GRP78 was predicted by HDOCK server. A more negative docking score means a more possible binding model and the two molecules would be very likely to bind when the confidence score is above 0.7. The results in Fig. 4B have displayed the high possibility of the binding between PLXNC1 and GRP78. At the same time, RT-qPCR and western blotting analyzed that GRP78 also displayed augmented expression in CHON-001 cells when treated with ascending doses of IL-1β (Fig. 4C). As expected, Co-IP assay testified the high affinity of PLXNC1 protein with GRP78 protein (Fig. 4D, E). Also, the increased GRP78 expression in CHON-001 cells following IL-1β treatment was declined by PLXNC1 depletion (Fig. 4F). Overall, PLXNC1 bond to GRP78 in IL-1β-challenged CHON-001 cells.

### GRP78 elevation counteracts the effects of PLXNC1 silencing on the viability and inflammation of IL-1β-exposed CHON-001 cells

**Fig. 5:**
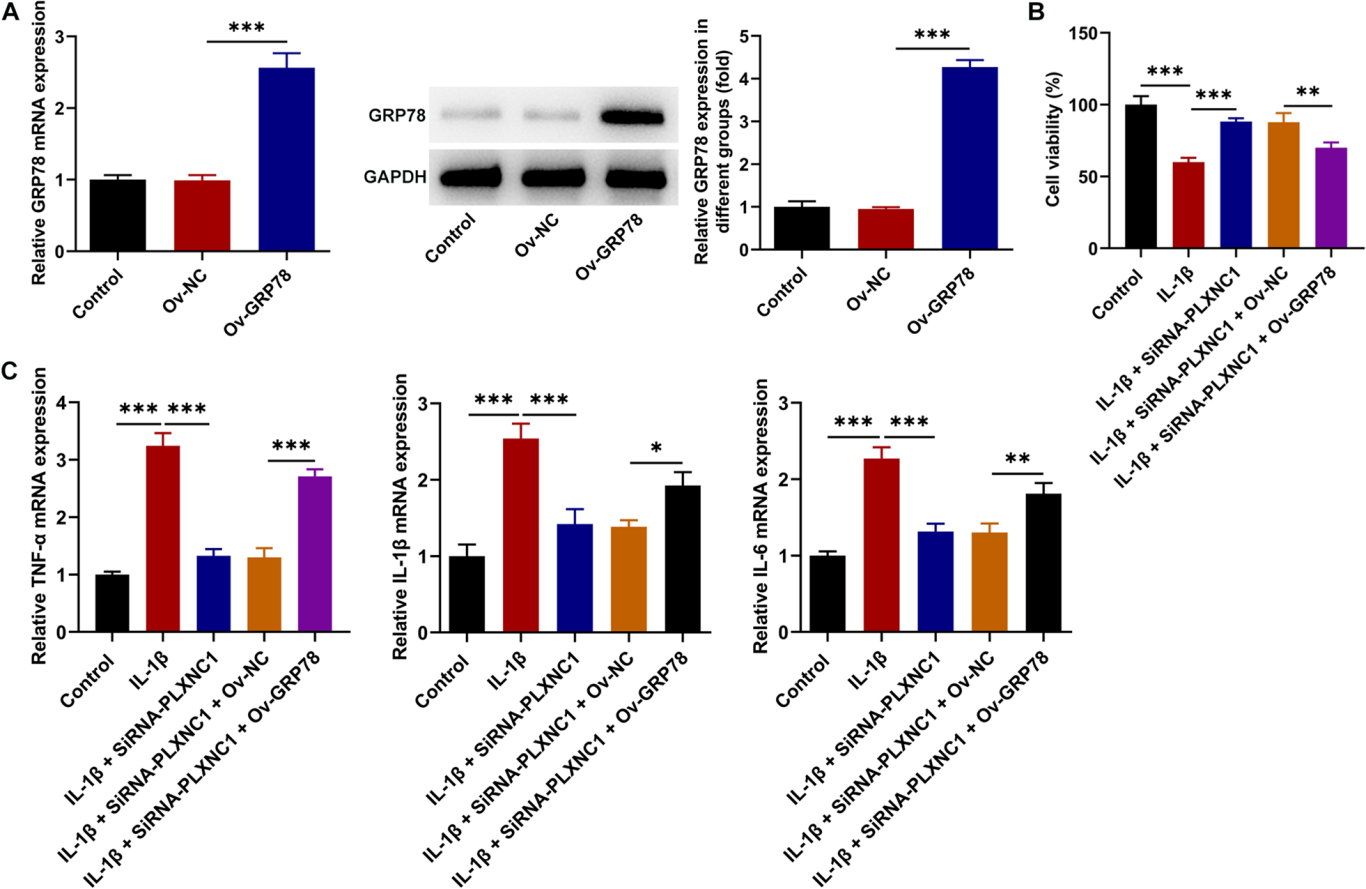
GRP78 elevation counteracts the effects of PLXNC1 silencing on the viability and inflammation of IL-1β-exposed CHON-001 cells. **A** and **B** RT-qPCR and western blotting tested the transfection efficiency of GRP78 overexpression plasmids. CHON-001 cells were treated by ascending doses of IL-1β (10 ng/ml) for 72 h and subsequently co-transfected with SiRNA-PLXNC1 and Ov-GRP78. **B** CCK-8 assay judged CHON-001 cell viability. **C** RT-qPCR examined the levels of inflammatory factors including TNFα, IL-1β and IL-6. *P < 0.05; **P < 0.01 and ***P < 0.001. *PLXNC1* plexin C1, *IL-1β* interleukin-1beta, *GRP78* glucose-regulating protein 78, *TNFα* tumor necrosis factor alpha, *IL-6* interleukin-6

To substantiate that PLXNC1 functioned in OA cell model via binding to GRP78, GRP78 was overexpressed following transduction of Ov-GRP78 plasmid (Fig. 5A). As CCK-8 demonstrated, the exacerbated viability of IL-1β-challenged CHON-001 cells imposed by PLXNC1 silencing was declined when GRP78 was up-regulated (Fig. 5B). Besides, PLXNC1 absence lowered the raised TNFα, IL-1β and IL-6 levels in CHON-001 cells exposed to IL-1β, which was then abolished by GRP78 elevation (Fig. 5C). In summary, PLXNC1 knockdown mitigated IL-1β-stimulated viability injury and inflammation in chondrocytes by down-regulating GRP78 expression.

### GRP78 elevation abolishes the impacts of PLXNC1 knockdown on the apoptosis and ECM degradation of IL-1β-exposed CHON-001 cells

**Fig. 6:**
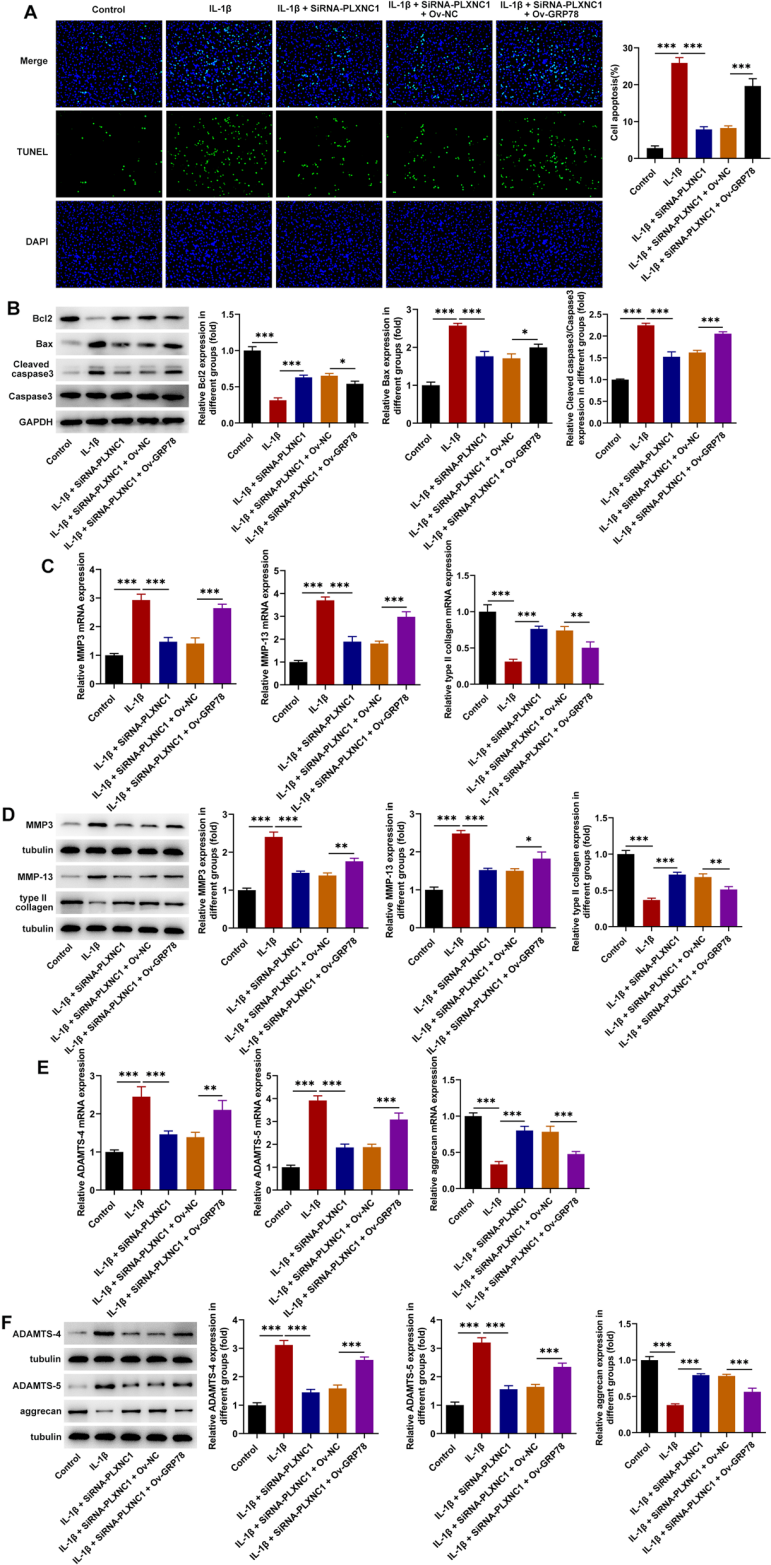
GRP78 elevation abolishes the impacts of PLXNC1 knockdown on the apoptosis and ECM degradation of IL-1β-exposed CHON-001 cells. CHON-001 cells were treated by ascending doses of IL-1β (10 ng/ml) for 72 h and subsequently co-transfected with SiRNA-PLXNC1 and Ov-GRP78. **A** TUNEL assay measured apoptosis. **B** Western blot analysis of the expression of apoptosis-associated proteins including Bcl-2, Bax, cleaved caspase 3 and caspase 3. **C**–**F** RT-qPCR and western blotting tested the expression of ECM degradation-associated proteins including MMP3, MMP-13, type II collagen, ADAMTS-4, ADAMTS-5 and aggrecan. *P < 0.05; **P < 0.01 and ***P < 0.001. *PLXNC1* plexin C1, *IL-1β* interleukin-1beta, *GRP78* glucose-regulating protein 78, *Bcl-2* B cell lymphoma-2, *Bax* Bcl-2-associated X, *MMP3* matrix metallopeptidase 3, *MMP-13* matrix metallopeptidase-13, *ADAMTS-4* a disintegrin and metalloproteinase with thrombospondin motifs type 4, *ADAMTS-5* a disintegrin and metalloproteinase with thrombospondin motifs type 5

Through TUNEL assay, it turned out that the inhibitory role of PLXNC1 silencing in IL-1β-induced CHON-001 cell apoptosis was abrogated by overexpression of GRP78 (Fig. 6A). As evidenced by western blotting, when GRP78 expression was elevated, the augmented Bcl-2 expression and the declined Bax, cleaved caspase 3/caspase 3 expression due to PLXNC1 absence in IL-1β-treated CHON-001 cells were all reversed (Fig. 6B). Similarly, PLXNC1 reduction cut down MMP3, MMP-13, ADAMTS-4, ADAMTS-5 expression and aggrandized type II collagen, aggrecan expression in IL-1β-challenged CHON-001 cells. However, following transduction of GRP78 overexpression plasmid, the impacts of PLXNC1 on the expression of ECM degradation-related factors were offset (Fig. 6C–F). Collectively, PLXNC1 interference eased IL-1β-stimulated apoptosis and ECM degradation in chondrocytes through reducing GRP78 expression.

### PLXNC1 interference blocks IL-1β-activated ERS signaling in CHON-001 cells through declining GRP78 expression

**Fig. 7:**
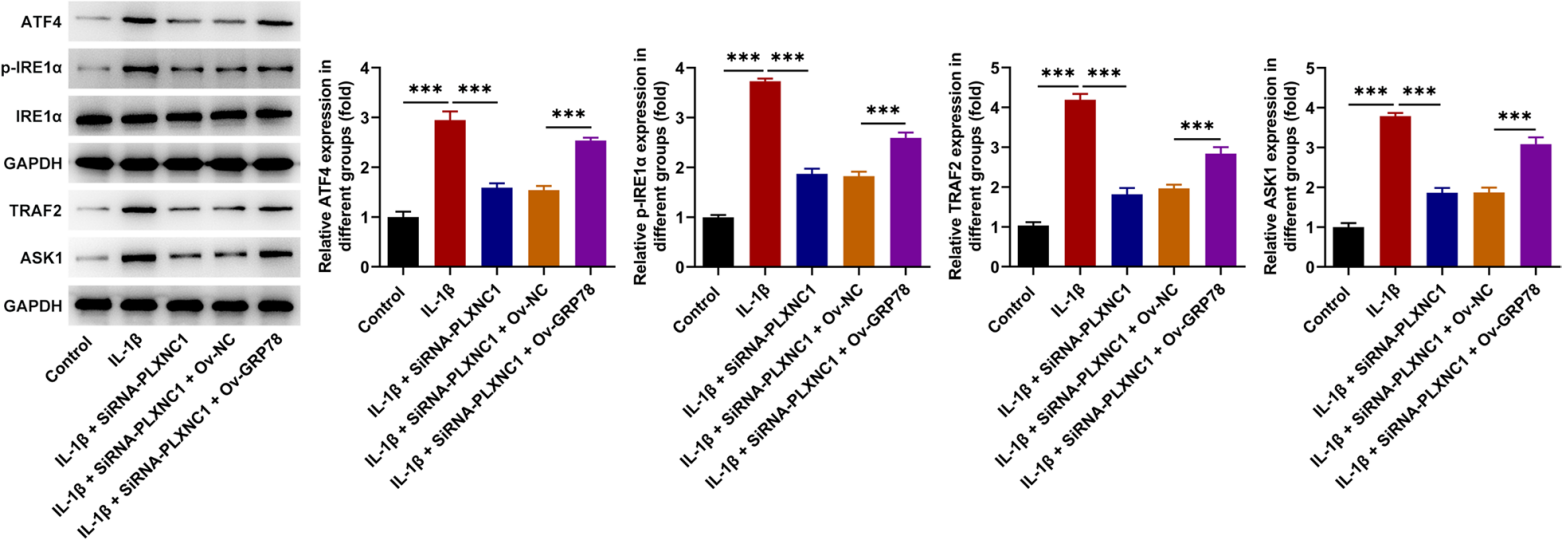
PLXNC1 interference blocks IL-1β-activated ERS signaling in CHON-001 cells through declining GRP78 expression. CHON-001 cells were treated by ascending doses of IL-1β (10 ng/ml) for 72 h and subsequently co-transfected with SiRNA-PLXNC1 and Ov-GRP78. Western blotting tested the expression of ERS signaling-associated proteins including ATF4, p-IRE1α/IRE1α, TRAF2 and ASK1. ***P < 0.001. *PLXNC1* plexin C1, *IL-1β* interleukin-1beta, *GRP78* glucose-regulating protein 78, *ATF4* activating transcription factor 4, *p-IRE1α* phosphorylated inositol requiring enzyme 1 alpha, *IRE1α* inositol requiring enzyme 1 alpha, *TRAF2* tumor necrosis factor receptor-associated factor 2, *ASK1* apoptosis signal-regulating kinase 1

Intriguingly, IL-1β exposure augmented ATF4, p-IRE1α/IRE1α, TRAF2 and ASK1 expression in CHON-001 cells. After PLXNC1 was silenced, ATF4, p-IRE1α/IRE1α, TRAF2 and ASK1 expression were all lessened in IL-1β-treated CHON-001 cells, which was then raised by GRP78 up-regulation (Fig. 7), suggesting that PLXNC1 knockdown suppressed GRP78 expression to inactivate ERS signaling in IL-1β-challenged CHON-001 cells.

## Discussion

OA is an autoinflammatory disease mediated by chondrocytes involving multiple factors [17]. Chondrocytes are important factors in maintaining the homeostasis of articular cartilage [18, 19]. During the process of OA, the accumulation of damaged organelles and degraded macromolecules in chondrocytes may destroy the balance of chondrocytes and result in the imbalance of cell synthesis and catabolism, leading to chondrocyte dysfunction [20, 21]. IL-1β, a pro-inflammatory cytokine expressed in the articular cartilage, peripheral blood and synovial tissues of OA patients, is capable of inducing the apoptosis and breaking the metabolic balance of chondrocytes during OA, hence being frequently applied to the induction of OA cell model [21–23]. Thence, making an inquiry into the cellular events of chondrocytes challenged with IL-1β during OA is of great significance to the treatment of OA.

PLXNC1 dysregulation is strongly associated with inflammatory reaction in human diseases, and all findings highlighted the protective role of PLXNC1 interference in inflammatory diseases [9, 24]. Based on GEO database, PLXNC1 expression was noted to be elevated in the cartilage tissues of patients with OA. Moreover, PLXNC1 exhibited dose-dependently fortified expression in CHON-001 cells exposed to IL-1β. Following knockdown of PLXNC1, the diminished CHON-001 cell viability imposed by IL-1β treatment was potentiated again. Inflammatory response remains a major physiological and pathological process of OA, and aberrant expression of inflammatory cytokines is viewed as a driver of OA, among which TNFα, IL-1β and IL-6 expression are reported to be closely related to the severity of OA [25–27]. Consistent with these findings, IL-1β exposure markedly elevated TNFα, IL-1β and IL-6 levels which were then declined by PLXNC1 absence, substantiating the anti-inflammatory property of PLXNC1 depletion in OA.

At the same time, inflammatory response-triggered excess apoptosis of chondrocytes may play a dominant role in the progression of OA [28]. Bax serves as a pro-apoptotic protein, while Bcl-2 functions as a suppressor of apoptosis, both of which inhibit each other and eventually mediate apoptosis via cleaved caspase 3 [29]. As expected, the apoptosis was aggravated, Bcl-2 expression was decreased and Bax, cleaved caspase 3 expression were augmented in IL-1β-treated CHON-001 cells. Under this condition, deficiency of PLXNC1 weakened the apoptotic ability of IL-1β-treated CHON-001 cells, which was also evidenced by the raised Bcl-2 expression and the lessened Bax, cleaved caspase 3 expression.

The degradation of ECM composed of proteoglycan and type II collagen is supported to be associated with joint damage in OA [30]. Under normal circumstances, chondrocytes may secrete a large amount of proteoglycan, which can be degraded by matrix metalloproteinases (MMPs) to maintain the metabolic balance of bone matrix [31]. Concurrently, pro-inflammatory cytokines in the injured chondrocytes may contribute to the excess generation of MMPs to result in ECM degradation [26, 32]. Additionally, ADAMTS-4 and ADAMTS-5 mainly take charge of the degradation of aggrecan, a key component of cartilage [33]. Through investigation, upon exposure to IL-1β, MMP3, MMP-13, ADAMTS-4, ADAMTS-5 expression were fortified and type II collagen, aggrecan expression were cut down in chondrocytes. More importantly, knockdown of PLXNC1 exerted opposite effects against IL-1β, which was manifested as descending MMP3, MMP-13, ADAMTS-4, ADAMTS-5 expression and ascending type II collagen, aggrecan expression.

Based on Biogrid database, the potential interacting partners of PLXNC1 were obtained. Also, the binding relationship between PLXNC1 and GRP78 was firstly confirmed by HDOCK server. Notably, recent studies have proposed that GRP78 expression is increased in OA cartilages and potentiates oxidative stress in chondrocytes [13, 14, 34]. Similarly, GRP78 expression was elevated in IL-1β-challenged CHON-001 cells and was lessened when PLXNC1 was down-regulated. The interaction between PLXNC1 and GRP78 in IL-1β-exposed CHON-001 cells was further testified by Co-IP assay. Moreover, overexpression of GRP78 countervailed the effects of PLXNC1 interference on the apoptosis, inflammatory response and ECM degradation in CHON-001 cells treated by IL-1β. ERS has also been well documented to be closely associated with the progression of OA [35–37]. Of note, GRP78 has been extensively valued as a regulator of ERS in chondrocytes in OA [14, 38]. As expected, it was discovered that IL-1β exposure augmented the expression of ERS-associated proteins including ATF4, p-IRE1α, TRAF2 and ASK1 in CHON-001 cells. After PLXNC1 was silenced, ATF4, p-IRE1α, TRAF2 and ASK1 expression were all lessened in IL-1β-treated CHON-001 cells, which were then raised by GRP78 up-regulation.

To sum up, PLXNC1 inhibition mitigated the apoptosis, inflammation as well as ECM degradation of IL-1β-insulted chondrocytes in OA by binding to GRP78 and down-regulating GRP78 expression. This study illuminated the role of PLXNC1 on the cellular events of chondrocytes in OA and revealed the possible binding relationship between PLXNC1 and GRP78, validating that PLXNC1 might be valued as an effective therapeutic target for OA.

## Data Availability

The analyzed data sets generated during the present study are available from the corresponding author on reasonable request.
